# Contextual processing in patients with schizophrenia

**DOI:** 10.1192/j.eurpsy.2023.2276

**Published:** 2023-07-19

**Authors:** M. Okruashvili, O.-H. Choung, D. Gordillo, M. Roinishvili, A. Brand, M. H. Herzog, E. Chkonia

**Affiliations:** 1 Tbilisi mental health centre, Tbilisi, Georgia; 2 École Polytechnique Fédérale de Lausanne, Lausanne, Switzerland; 3 Ivane Beritashvili Centre of Experimental Biomedicine; 4 Tbilisi State Medical University, Tbilisi, Georgia

## Abstract

**Introduction:**

Patients with schizophrenia have deficits in contextual vision. However, results are often very mixed. In some paradigms, patients do not take the context into account and therefore act more veridically than healthy controls. In other paradigms, context impairs performance in patients more strongly than in healthy controls. These mixed results may be explained by differences in paradigms, as well as by small or biased samples, given the large heterogeneity of the disease.

**Objectives:**

To understand if there are general contextual deficits in schizophrenia.

**Methods:**

17 schizophrenia patients and 16 age-matched controls were tested with a combined crowding and uncrowding paradigm.

**Results:**

Schizophrenia patients show qualitatively similar crowding performance as controls. In the uncrowding condition, however, patientsimproved less than controls. We suggest that performance in the various paradigms depends on idiosyncratic aspects of the paradigm in addition to the heterogeneity of the disease.

**Conclusions:**

There are no general impaired mechanisms in schizophrenia. Deficits depend strongly on idiosyncrasies of the specific stimuli.

**Disclosure of Interest:**

None Declared

